# Mechanochemical fabrication and properties of CL-20/RDX nano co/mixed crystals

**DOI:** 10.1039/c8ra04122a

**Published:** 2018-10-04

**Authors:** Xiaolan Song, Yi Wang, Shanshan Zhao, Fengsheng Li

**Affiliations:** School of Environment and Safety Engineering, North University of China Taiyuan 030051 China songxiaolan00@126.com; School of Materials Science and Engineering, North University of China Taiyuan 030051 China wangyi528528@aliyun.com; School of Chemical Engineering, Nanjing University of Science and Technology Nanjing 210094 China

## Abstract

By milling 2,4,6,8,10,12-hexanitro-2,4,6,8,10,12-hexaazaisowurtzitane (CL-20) and hexahydro-1,3,5-trinitro-1,3,5-triazine (RDX) together, a nano CL-20/RDX co/mixed crystal explosive with a mean particle size of 141.6 nm is prepared from the raw materials, and the co/mixed crystals are characterized using scanning electron microscopy (SEM), transmission electron microscopy (TEM), X-ray diffraction (XRD), Raman spectroscopy, infrared (IR) spectroscopy, X-ray photoelectron spectroscopy (XPS), differential scanning calorimetry (DSC) and thermal-infrared spectrometry online (DSC-IR) technology; furthermore, the impact, friction and thermal sensitivity of the samples are tested. The results show that after milling, the morphology of the co/mixed crystal explosive is near-spherical, and the particle size reveals a normal distribution. The milled sample showed the same molecular structure and surface elements as the raw materials, but the XRD test shows that CL-20/RDX has a new crystal phase and the Raman and IR spectra gave a supplementary confirmation for the existence of a cocrystal phase in the milled sample. The activation energy of the thermal decomposition of CL-20/RDX is 206.49 kJ mol^−1^ higher than that of raw RDX. DSC-IR analysis showed that the thermolysis of CL-20/RDX produces a large amount of CO_2_ and N_2_O and a small amount of H_2_O, NO_2_ and NO. The mechanical sensitivity of CL-20/RDX is very low. In impact sensitivity tests with a 5 kg hammer, the special height (*H*_50_) is 51.43 cm, which is higher than the values of 36.43 cm for raw CL-20 and 9.78 cm for raw RDX. In the friction sensitivity tests, the explosion probability (*P*) is 56%; however, the thermal sensitivity of CL-20/RDX is higher than that of the raw materials, with its 5 s burst point being only 243.51 °C.

## Abstract 2

A nano CL-20/RDX co/mixed crystal explosive with mean size of 141.6 nm is prepared by mechanical milling method. Its structure is characterized and thermal decomposition is investigated. Additionally, its impact, friction and thermal sensitivities are tested.

## Graphical abstract

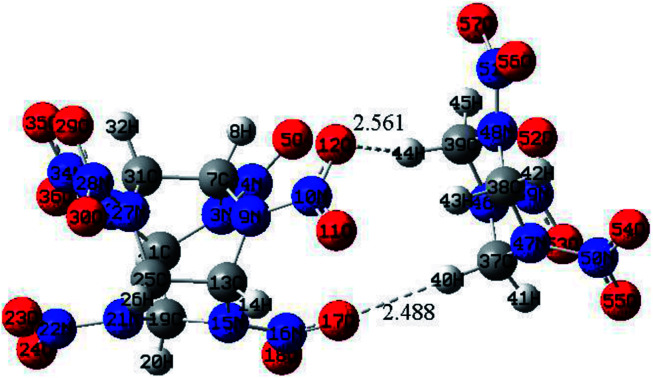

## 1 Introduction

Energetic materials are usually defined as compounds storing large amounts of energy which can burn or explode rapidly under certain external and environmental conditions.^1,2^ However, high-energy explosives often have problems of low safety and high cost, limiting their further development. In recent years, improvements in insensitivity without changing the performance of explosives have been made with 4 phases (*i.e.*, surface coating, spheroidization, nanocrystallization and cocrystallization). According to current research in this field, nanocrystallization and cocrystallization are superior to surface coating and spheroidization in terms of the enhancement of the insensitivity of energetic materials.^3–5^ In our previous studies, using mechanical milling, we fabricated many kinds of nanoexplosives and investigated their properties.^6–9^ These nanoexplosives were of particle sizes of 100–200 nm. Furthermore, nanoexplosives show much lower sensitivities than microexplosives.

Moreover, cocrystallization is also a good method to improve the performance of explosives. As early as in 1978, the 1,3,5,7-tetranitro-1,3,5,7-tetrazocane/ammonium perchlorate (HMX/AP) cocrystal was prepared by a decompression solvent evaporation method in which the hydrophobicity of AP was substantially improved.^10^ Subsequently, researchers prepared 2,4,6,8,10,12-hexanitro-2,4,6,8,10,12-hexaazaisowurtzitane/2,4,6-trinitrotoluene (CL-20/TNT),^11^ CL-20/HMX,^12–14^ 2,4,6,8,10,12-hexanitro-2,4,6,8,10,12-hexaazaisowurtzitane/dinitrobenzene (CL-20/DNB),^15^ 1,3,5-triamino-2,4,6-trinitrobenzene/1,3,5,7-tetranitro-1,3,5,7-tetrazocane (TATB/HMX),^16^ and other cocrystal explosives^17–19^ and reduced the sensitivity of explosives to a certain extent. For example, the nano CL-20/HMX explosive was prepared with the spray drying method.^20^ The micron-scale morphology of the cocrystal was spherical with size of 0.5–5 μm, of which the mechanical sensitivities were obviously lower than those of the raw materials. However, the yield of traditional cocrystallization technology (solvent evaporation) is very small.^21–23^ It is difficult to prepare 1 g of fine cocrystal in each pot by the solvent evaporation method. The mechanical milling method is a novel cocrystallization technology that can be used to solve the problem of low yield. In fact, the mechanical milling method has already been used in the fabrication of drug cocrystals.^24,25^ After milling, different drug crystals were incorporated with the formed cocrystals. In addition, the mechanical milling method was also used to prepare the explosive. For example, ultrafine HMX/TATB fabricated by using this method.^26^ Therein, the prepared HMX/TATB particles were sizes of 100–300 nm, and the mechanical sensitivity of the prepared particles is lower than that of raw HMX.

Overall, if the mechanical milling method was combined with nanocrystallization and cocrystallization, the sensitivity of high-energy explosives would be further reduced *i.e.* their safety would further be improved.

## 2 Experimental

### 2.1 Materials

Raw CL-20 was purchased from the Beijing Institute of Technology (Beijing City, P. R. China); raw RDX was purchased from Gansu Yinguang Chemical Co., Ltd. (Baiyin City, Gan-Su Province, P. R. China); ethyl alcohol was purchased from Tianjin Guangfu Chemical Co., Ltd. (Tianjin City, P. R. China); zirconia balls (*ϕ* = 0.3 mm) were purchased from Shandong Zibo Yubang Ceramics Co., Ltd. (Zibo City, Shandong Province, P. R. China).

### 2.2 Fabrication

Raw CL-20 (5 g), raw RDX (5 g), zirconia balls (200 g), ethyl alcohol (50 mL), and distilled water (50 mL) were put into an aluminum oxide milling jar simultaneously. After sealing, the aluminum oxide milling jar was installed in an MITR planetary ball mill (YXQM-1L), with a rotation speed set at 350 rpm. After 6 hours of milling, the jar was opened, and the material was removed. Then, the nano CL-20/RDX co/mixed crystal powder was separated from the zirconia balls using an ultrasonic cleaner. After freeze-drying, the nano CL-20/RDX co/mixed crystal powder was obtained. This ball mill can hold 4 milling jars at a time. Thus, 40 g of nano CL-20/RDX co/mixed crystal powder can be prepared with the existing process.

### 2.3 Characterization and tests

The morphology was observed with field-emission scanning electron microscopy (SEM, JEOL JSM-7500) and transmission electron microscopy (TEM, Philips-Tecnai). X-ray diffraction (XRD, Bruker Advance D8) was used to investigate the phase of the samples. Cu Kα radiation at 40 kV and 30 mA was chosen for the test. The structure of the samples was analyzed using a JY-HR800 confocal Raman spectrometer manufactured by Jobin Yvon, France. IR analysis was performed on an American Thermo Fisher Scientific Nicolet 6700 infrared spectrometer using potassium bromide tablets; elemental analysis was performed using X-ray photoelectron spectroscopy (XPS, Ulvac-PhiPHI-5000, Japan); thermal analysis was conducted with differential scanning calorimetry (DSC, TA Model Q600) at heating rates of 5, 10, 15, and 20 °C min^−1^; DSC-IR analysis was carried out at a heating rate of 10 °C min^−1^ using a thermal analysis system (TG/DSC, Mettler Toledo) coupled with a Fourier transform infrared spectrometer.

The impact sensitivity of the samples was tested with an HGZ-1 impact instrument. The special height (*H*_50_) represents the height from which a 5.0 kg drop-hammer results in an explosive event in 50% of the trials. In each determination, 25 drop tests were performed to calculate the *H*_50_, with each lot tested three times to obtain a mean value and a standard deviation (*S*_dev_). The testing conditions are as follows: sample mass, 35 mg; temperature, 10–35 °C; and humidity, ≤80%. The friction sensitivity of the samples was tested with a WM-1 friction instrument. In each determination, 25 samples were tested and the explosion probability (*P*, %) was obtained. Each lot was tested three times to obtain a mean value and a standard deviation (*S*_dev_). Experimental conditions are shown as follows: pendulum weight, 1.5 kg; swaying angle, 80°; pressure, 2.45 MPa; and sample mass, 20 mg. Thermal sensitivity tests (*i.e.*, 5 s bursting point tests) were carried out in compliance with GJB772A-97 method 606.1.

To theoretically investigate the intermolecular interactions of CL-20/RDX, the structure of CL-20/RDX was optimized at the DFT-B3LYP/6-31+G using Gaussian 09.^27^ Multiwfn^28^ was used to analyze the molecular surface electrostatic potential.

## 3 Results and discussion

### 3.1 Theoretical calculation

**Fig. 1:**
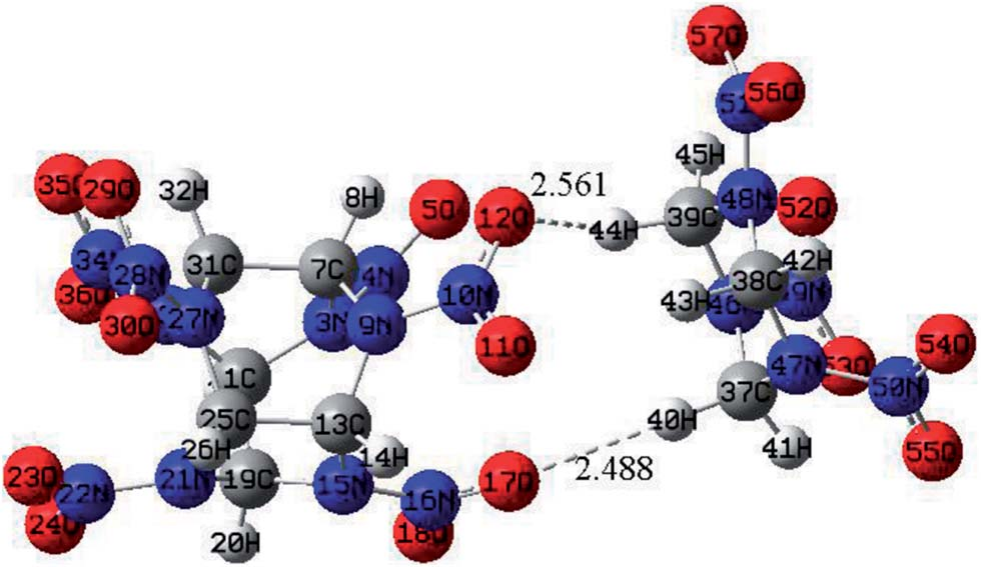
Optimized structure of CL-20/RDX at the DFT-B3LYP/6-31+G level.

**Fig. 2:**
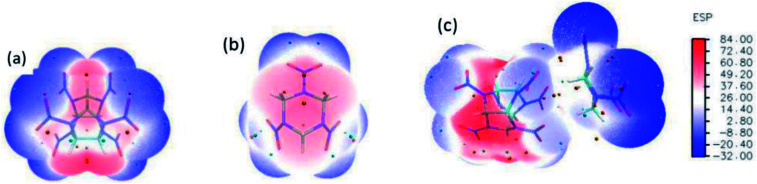
Molecular electrostatic potential distribution of the structures of explosives: (a) CL-20, (b) RDX, (c) CL-20/RDX.

The optimized structure of CL-20/RDX at the DFT-B3LYP/6-31+G level is shown Fig. 1. It can be found that the O atom of N–O in CL-20 interacts with the H atom of C–H in RDX. Obviously, intermolecular interactions primarily occur due to C–H⋯O bonds. The H⋯O bond lengths are 2.561 Å and 2.488 Å, respectively. These values are within the length scope of the hydrogen bonds.^29^ To obtain the molecular electrostatic potential distribution of structures of CL-20, RDX and their cocrystals, these structures were analyzed by the Multiwfn program. The results are shown in Fig. 2 from which it can be seen that the blue portion refers to the negative electrostatic region, the red portion refers to the positive electrostatic region, and the white portion refers to the neutral region. For the CL-20 and RDX molecules, negative electrostatic regions are mainly distributed on two O atoms in the –NO_2_ group, and positive electrostatic regions are mainly distributed on –CH_2_–, –CH_3_ and –H. However, there are different phenomena in the CL-20/RDX cocrystal. At the junction of the molecules, the blue area disappears into white, meaning that the charge is changed and tends to be neutral. This is because the surface of electrostatic potential of the O atoms in the –NO_2_ group in the CL-20 molecule coincides with the surface of the electrostatic potential of C–H in the RDX molecule and the positive and negative electrostatic regions overlap, resulting in a change in electrostatic potential at the junction. This indicates that there are intermolecular forces in the CL-20/RDX cocrystal, which is consistent with the structural analysis results.

### 3.2 Morphology and structure

**Fig. 3:**
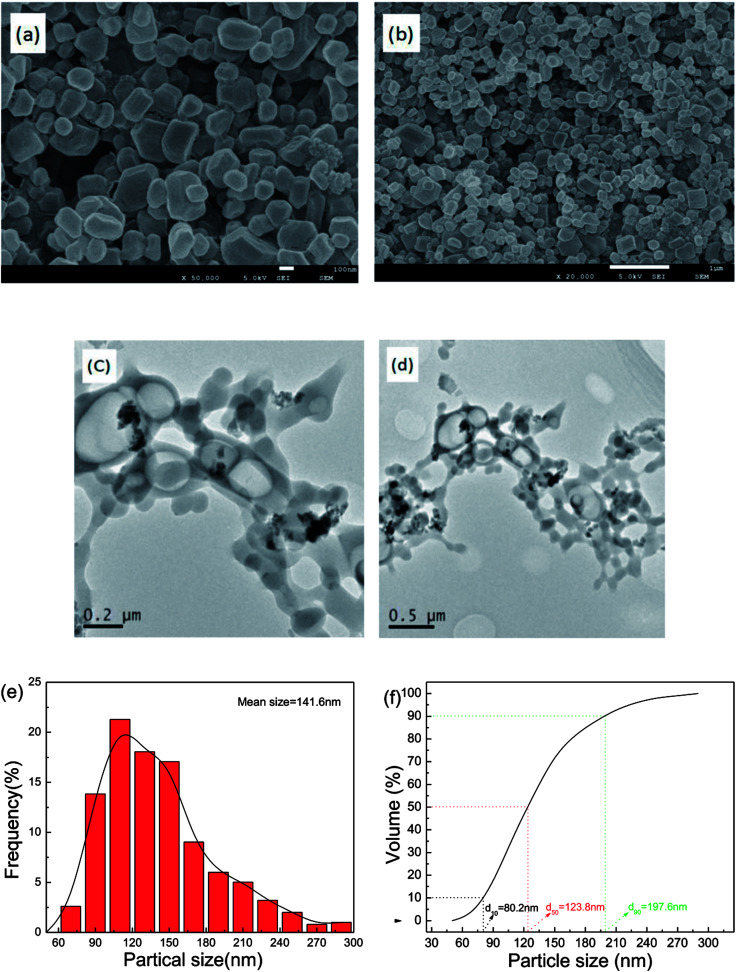
SEM and TEM images and particle size distribution of nano CL-20/RDX co/mixed crystals.

The morphology of as-prepared samples was probed by SEM and TEM analysis, with the results illustrated in Fig. 3. In Fig. 3a and b, the fine particles obtained are near-spherical and of uniform size. Fig. 3c and d show that the TEM images of as-prepared samples and the TEM images of the particle morphology and size agree with the SEM images. Approximately 500 particles were counted in Fig. 3b by Nano Measurer 1.2 software. Then, the frequency distribution curve and the volume distribution curve were obtained and are shown in Fig. 3e and f. It is clear that the particle size distribution generally exhibits a normal distribution with a mean size of 141.6 nm. The volume distribution curve reveals that a majority of the milled particles have diameters less than 197.6 nm (*d*_90_ = 197.6 nm), with a median diameter of 123.8 nm (*d*_50_ = 123.8 nm).

**Fig. 4:**
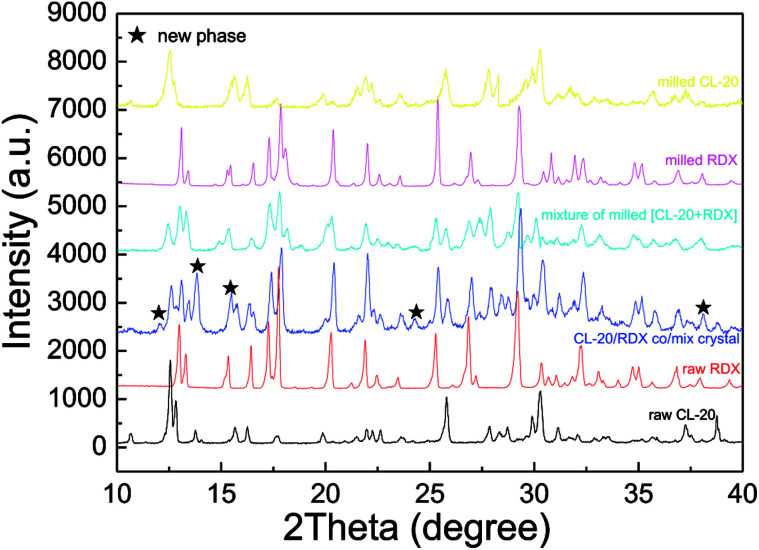
XRD pattern of samples.

To investigate the sample phases of raw CL-20, raw RDX, and CL-20/RDX after milling, XRD analysis was performed, and the patterns are shown in Fig. 4. Compared with the patterns of raw CL-20 and raw RDX, new diffraction peaks appeared on the patterns of CL-20/RDX, such as 12.0°, 24.3° and 34.8°. Additionally, some peak positions of CL-20/RDX did not move, but the peak intensities changed. These changes illustrate that the as-prepared samples did not result in simple mixing of CL-20 and RDX, but they interacted to form a new state of co/mixed crystals. To further verify this conclusion, raw RDX and raw CL-20 were also milled respectively and the sample “milled RDX”, “milled CL-20”, and “mixture of milled [CL-20 + RDX]” were analyzed by XRD analysis. The sample “mixture of milled [CL-20 + RDX]” was prepared with simply blending the “milled CL-20” and the “milled RDX”. The results were also illustrated in Fig. 4. It can be found that XRD patterns of milled CL-20 and milled RDX are same to the patterns of raw CL-20 and raw RDX. Compared with the XRD patterns of “milled CL-20” and “milled RDX”, there are no new diffraction peak presenting in XRD pattern of “mixture of milled [CL-20 + RDX]”. However, contrasting the XRD patterns of “CL-20/RDX co/mixed crystal” and “mixture of milled [CL-20 + RDX]”, it is clear that new diffraction peaks exist in the pattern of “CL-20/RDX co/mixed crystal”. The result further confirms the formation of some new phase(s) in the sample of “CL-20/RDX co/mixed crystal”.

**Fig. 5:**
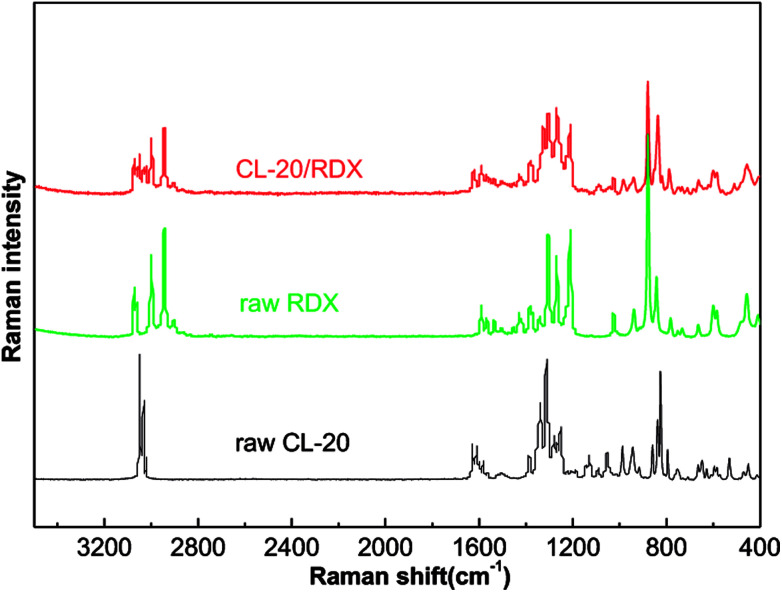
Raman spectra of the samples.

Raman tests of the samples are used to explore the cause of co/mixed crystal formation, as shown in Fig. 5. Table 1 lists the assigned major bands of the Raman spectra of the samples. It can be seen that most of the Raman peaks of CL-20 and RDX shifted in the co/mixed crystal Raman spectrum. The asymmetric –NO_2_ stretching vibration in CL-20 is at 1628.2 cm^−1^ and 1562.4 cm^−1^, but they shifted to 1624.6 cm^−1^ and 1558.4 cm^−1^ in CL-20/RDX. A similar phenomenon was observed with the peak at 3000.7 cm^−1^, representing symmetric the –CH_2_ stretching vibrations in RDX, which shifted to 2997.7 cm^−1^. Interestingly, some peaks disappeared. They represent the symmetric –NO_2_ stretching vibration in CL-20 at 1280.9 cm^−1^ to 1248.6 cm^−1^. This is due to the formation of C–H⋯O hydrogen bonds between –NO_2_ (CL-20) and –CH_2_ (RDX).

**Table 1:** Assignment of the major bands of the Raman spectra of the samples

Assignment	Raw CL-20	Co/mixed crystal	Raw RDX	Assignment
		3073.8	3076.8	C–H stretching vibration
		3062.0	3065.0	–CH_2_ stretching vibration
–CH_2_ stretching vibration	3050.4	3050.9		
	3031.7	3030.2		
		2997.7	3000.7	
		2942.3	2948.2	C–H stretching vibration
		2903.9	2906.9	
Asymmetric –NO_2_ stretching vibration	1628.2	1624.6		
	1608.2	1609.9		
	1598.4	1591.4	1589.9	–NO_2_ stretching vibration
	1579.0	1569.3	1571.6	
	1562.4	1558.4		
		1461.2	1454.4	–CH_2_ deformation vibration
		1422.9	1430.6	
C–H and –CH_2_ deformation vibration	1389.4	1380.8	1383.8	
	1342.7	1351.1	1345.2	
	1309.2	1307.3	1310.2	–CH_2_ and N–N deformation vibration
Symmetric –NO_2_ stretching vibration	1280.9			
	1248.6			
		1209.4	1210.0	C–N stretching vibration
Asymmetric C–H stretching vibration	1129.4	1109.3		
N–N stretching vibration	1059.6	1055.5		
		1029.3	1026.4	C–N stretching vibration
C–C stretching vibration	914.4	912.0		
		880.0	881.2	C–N stretching vibration
Ring stretching vibration	840.8	837.8	844.0	
Ring deformation vibration	823.9	819.5		
Symmetric –NO_2_ stretching vibration	793.7	789.3		
		788.8	782.7	–NO_2_ deformation vibration

**Fig. 6:**
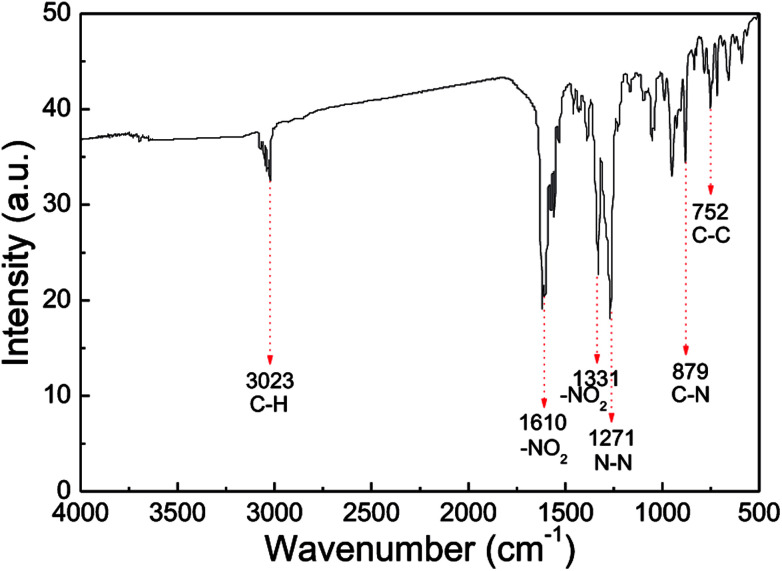
IR spectra of samples.

IR analysis was employed to identify the molecular structure of the nano CL-20/RDX co/mixed crystal, and the results are displayed in Fig. 6. In the IR spectrum of the nano CL-20/RDX co/mixed crystal, the peak at 3023 cm^−1^ reflects the stretching vibrations of C–H. The peaks at 1610 cm^−1^ and 1331 cm^−1^ represent the asymmetric stretching vibrations of –NO_2_ and the symmetric stretching vibrations of –NO_2_, respectively. The peak at 1271 cm^−1^ refers to the stretching vibrations of N–N. The peaks at 879 cm^−1^ and 752 cm^−1^ correspond to the stretching vibrations of C–N and C–C, respectively. These results mean that the functional groups in the as-prepared sample structures are consistent with those in the raw material. Overall, the molecular structures of CL-20/RDX were not changed after the milling process.

**Fig. 7:**
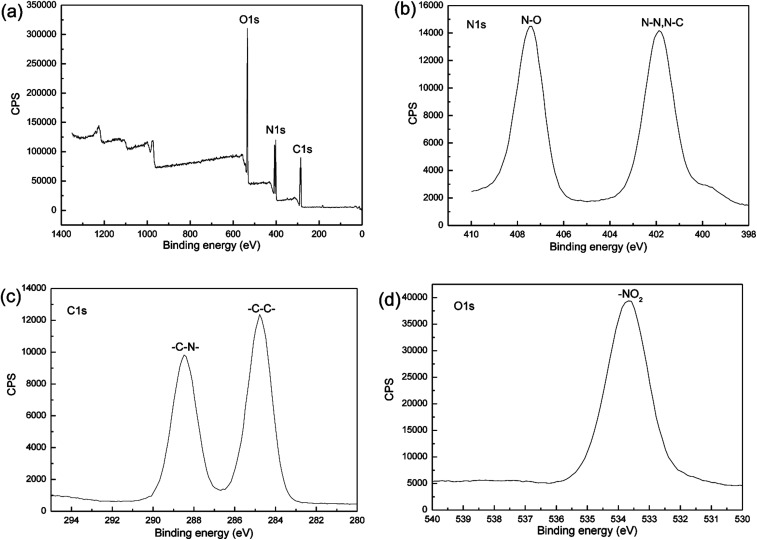
XPS spectrum of the nano CL-20/RDX co/mixed crystal.

In addition, XPS analysis was conducted to confirm whether the milling process introduces impurities into the sample. The XPS spectra of the nano CL-20/RDX co/mixed crystal are illustrated in Fig. 7. In both spectra, the peak in Fig. 7a is associated with O 1s, C 1s and N 1s, and the peak in Fig. 7b is associated with N 1s. The excitation of the N 1s electron results in the two curves, *i.e.*, ammonia nitrogen (N–N or N–C) and nitrate nitrogen (N–O). Because of the different electronegativity of C, N and O elements (the electronegativity order is O > N > C), the corresponding two kinds of nitrogen atoms have different chemical shifts. The O 1s peak allows for the determination of the effect of the nitrate nitrogen (N–O) on the electron excitation of O 1s. The C 1s peak illustrates the influence of the hydrogen atoms in –CH_2_ group and the ammonium nitrogen on electron excitation of C 1s. No other elements were detected in the spectrum, an indication that the sample was not polluted.

### 3.3 Thermal analysis

**Fig. 8:**
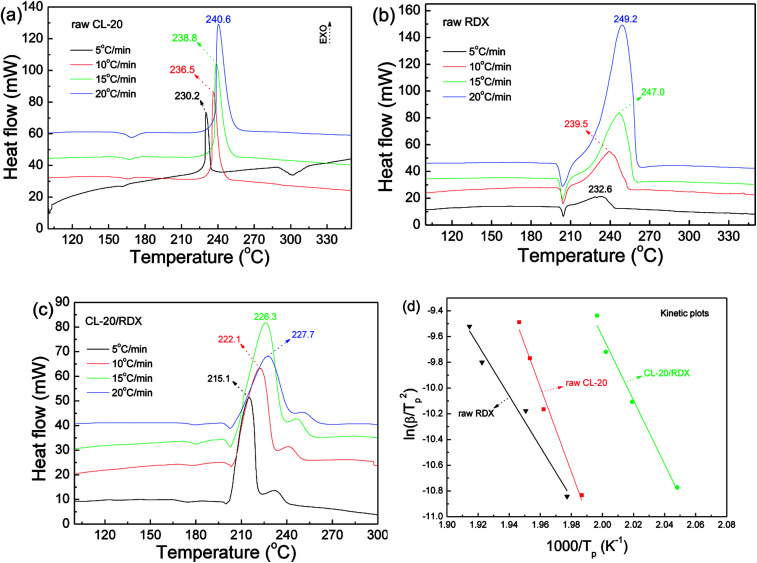
DSC traces of the samples.

Thermal analysis was conducted, and the DSC traces are shown in Fig. 8. The DSC traces of raw CL-20 have a small endothermic peak at 160 °C, corresponding to the phase transformation of CL-20. When the temperature exceeds 230 °C, a distinct exothermic peak appearing on the DSC traces reflects the thermal decomposition of CL-20. Furthermore, there was a normal phenomenon presented in the DSC traces of raw CL-20, *i.e.*, the decomposition heat increased as the heating rate increased. The DSC traces of raw RDX reveal that there were neither exothermic nor endothermic processes before a temperature of 200 °C. When the temperature reached approximately 203 °C, there were small endothermic peaks followed by the emergence of large exothermic peaks, indicating the melting of RDX. Fig. 8c shows the DSC traces of nano CL-20/RDX co/mixed crystal, and it is obvious that CL-20/RDX was very stable before a temperature of 200 °C. A small endothermic peak appears at a temperature of approximately 203 °C, referring to the melting process of the raw RDX. Subsequently, two exothermic peaks appeared, representing the thermal decomposition of CL-20/RDX and RDX, respectively. In fact, there is still a small amount of free RDX in co/mixed crystal explosives. However, it can be seen that the thermal decomposition heat of CL-20/RDX decreased, which indicates that two explosive molecules are not simply mixed, and most of the RDX and CL-20 forms a co/mixed crystal explosive. Probing the kinetic and thermodynamic parameters is very important for mastering the thermolysis properties of energetic materials. Herein, in order to further study the thermal decomposition process of nano CL-20/RDX co/mixed crystal explosives, the thermodynamic and kinetic parameters of thermal decomposition were calculated by using eqn (1)–(5). The results are shown in Fig. 8d and Table 2.1
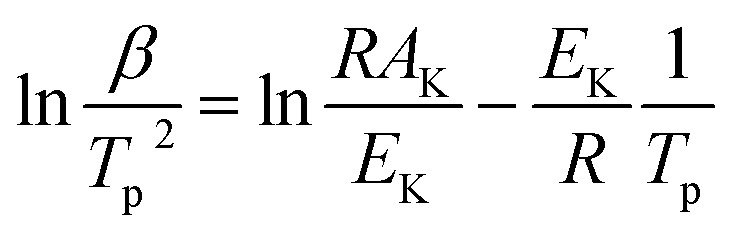
2
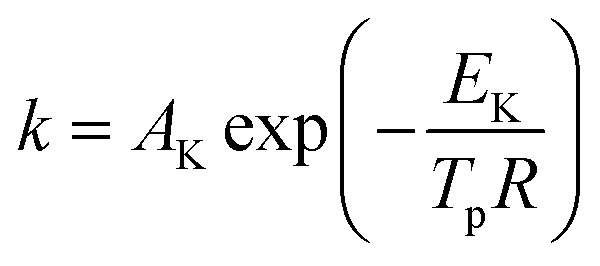
3
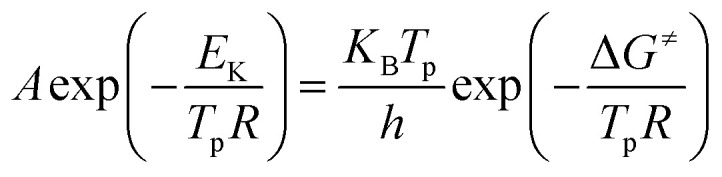

4 Δ*H*^≠^ = *E*_K_ − *RT*_p_

5 Δ*G*^≠^ = Δ*H*^≠^ − *T*_p_Δ*S*^≠^

where *T*_p_ is the peak temperature in the DSC trace with a heating rate of 15°C min^−1^; *K*_B_ and *h* are Boltzmann's (*K*_B_ = 1.381 × 10^−23^ J K^−1^) and Planck's constants (*h* = 6.626 × 10^−34^ J s^−1^), respectively; *β* is the heating rate; *k* is thermal decomposition rate constant; *E*_K_ and *A*_K_ are the activation energy and pre-exponential factor calculated by the Kissinger equation; Δ*H*^≠^ is the thermal decomposition activation enthalpy, J mol^−1^; Δ*G*^≠^ is the thermal decomposition activation free energy, J mol^−1^; and Δ*S*^≠^ is the thermal decomposition activation entropy, J mol^−1^ K^−1^.

**Table 2:** Thermodynamic and kinetics parameters derived from the DSC traces

Samples	*T*_p_ (K)	Thermodynamics			Kinetics		
		Δ*H*^≠^ (kJ mol^−1^)	Δ*G*^≠^ (kJ mol^−1^)	Δ*S*^≠^ (J mol^−1^ K^−1^)	*E*_K_ (kJ mol^−1^)	ln *A*_K_	*k* (s^−1^)
Raw CL-20	511.95	269.74	125.00	282.72	274.00	65.00	1.88
Raw RDX	520.15	160.26	129.03	60.04	164.59	38.24	1.20
CL-20/RDX	499.45	202.33	122.52	159.80	206.49	50.19	1.60

The Kissinger equation (eqn (1)) and Arrhenius equation (eqn (2)) were enlisted to calculate the activation energy (*E*_K_), pre-exponential factor (*A*_K_), and rate constant (*k*). The results indicate that the activation energies of raw CL-20 and raw RDX are 274.00 kJ mol^−1^ and 164.59 kJ mol^−1^, respectively. The activation energy of CL-20/RDX is 206.49 kJ mol^−1^, which is 41.9 kJ mol^−1^ higher than the *E*_K_ of the raw RDX but 67.51 kJ mol^−1^ lower than that of raw CL-20. For the rate constant *k*, CL-20/RDX is larger than raw RDX and smaller than CL-20. To gain a deeper insight into their thermal decomposition, eqn (3)–(5) were used to obtain the activation enthalpy (Δ*H*^≠^), activation entropy (Δ*S*^≠^), and activation free energy (Δ*G*^≠^). The physical meaning of Δ*H*^≠^ is the energy that the molecules absorb to change from the common state to the activated state, and thus the value of Δ*H*^≠^ is much closer to that of *E*_K_ for each sample. In this paper, we found that Δ*H*^≠^ of CL-20/RDX is more than that of raw RDX and less than that of raw CL-20, meaning activation of CL-20/RDX needs more energy than raw RDX. Δ*G*^≠^ is the change in Gibbs energy that reaches the activated state of the explosive molecules. For all samples, the values of Δ*G*^≠^ were positive numbers, meaning that thermal decomposition of the explosives cannot occur spontaneously. Therefore, the energetic materials were in a very stable state unless sufficient energy was introduced.

**Fig. 9:**
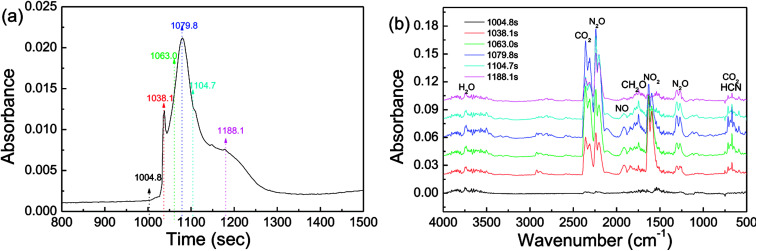
DSC-IR spectra of CL-20/RDX: (a) the DSC curve in DSC-IR analysis; (b) IR spectra of gas products intercepted at different times.

Investigation of decomposition products is of importance to provide deeper insight into thermolytic properties of energetics. *Via* DSC-IR analysis, the products of the thermal decomposition of CL-20/RDX were detected, and the results are shown in Fig. 9. In Fig. 9a, there are two relatively sharp absorption peaks between 1004.8 s and 1188.1 s, recording the decomposition of CL-20/RDX. By analyzing the IR spectra in Fig. 9b, the main gas products were identified as follows. Strong peaks in the range from 2310–2360 cm^−1^ revealed the existence of a large amount of CO_2_. Then, nitrogen oxides were also detected, in which the peaks located at 2200–2240 cm^−1^ correspond to a large amount of N_2_O gas, and the peak at 1630 cm^−1^ indicate NO_2_ gas. It is generally believed that the chief reason for this phenomenon in the IR spectra is that the split radical of ·NO_2_ (from N–NO_2_) reacted with the –CH– fragment (from the carbon chain). The peak at 1749 cm^−1^ reflected the fragment of CH_2_O. The peak at 669 cm^−1^ implies the existence of HCN resulting from the rupture of the N–N bond in nitroamine molecules. Actually, the thermal decomposition of explosives is related to the cracking of the molecule, independent of the crystal phase, and it does not alter the structure of the molecule whether it is a cocrystal or a mixed crystal. As a rule, the thermolysis of nitroxide and nitroamine explosives begins with the cracking of C–NO_2_ or N–NO_2_ bonds in the molecule. In this paper, we find that a large amount of CO_2_ gas rather than CO appeared on the IR spectra, indicating that at relatively low temperatures, decomposition proceeded nearly to completion and fully released its chemical energy.

### 3.4 Sensitivities

To evaluate the safety of CL-20/RDX, tests on the impact, friction and thermal sensitivities were performed, with the results presented in Table 3. Raw CL-20 and raw RDX are of the mean size of 59.21 μm and 88.03 μm, respectively. The sample “CL-20/RDX blend” is a simple mixture of raw CL-20 and raw RDX. After milling, the impact and friction sensitivities of the nano CL-20/RDX co/mixed crystal significantly decreased. As seen in Table 3, the special heights (*H*_50_) of raw CL-20 and raw RDX were 15.0 cm and 41.65 cm, respectively, while the special height of the nano co/mixed crystal explosive was 51.43 cm, which was higher than the 36.43 cm value of raw CL-20 and 9.78 cm of raw RDX. In addition, the explosion probability of friction (*P*) was 56% lower than that of raw CL-20, raw RDX and their blends. However, the thermal sensitivity test results are contrary to the mechanical sensitivity test results. The 5 s burst point (*T*_5s_) of CL-20/RDX is only 243 °C, which means that it has the highest thermal sensitivity compared to those of the raw materials. CL-20/RDX is not suitable for use as a heat-resistant explosive.

**Table 3:** Results of sensitivity tests for the samples

Samples	Impact sensitivity		Friction sensitivity		Thermal sensitivity
	*H*_50_ (cm)	*S*_dev_	*P* (%)	*S*_dev_	*T*_5s_ (°C)
Raw CL-20	15.00	3.86	100%	0	283.57
Raw RDX	41.65	7.11	74%	3.34	301.20
CL-20/RDX blend	30.18	4.25	100%	0	—
CL-20/RDX co/mixed crystal	51.43	5.71	56%	2.85	243.51

## 4 Conclusions

The mechanical milling method is used to prepare nano CL-20/RDX co/mixed crystal explosives with a mean particle size of 141.6 nm. Before and after milling, the surface elements and molecular structures of CL-20/RDX do not change compared to those of raw CL-20 and raw RDX. However, new crystal phases were presented in the XRD pattern. Raman spectra imply that the formation of CL-20/RDX cocrystals originates from C–H⋯O hydrogen bonds between –NO_2_ and –CH_2_. The DSC analysis is employed to probe the thermolytic properties of the samples. CL-20/RDX has a high activation energy and activation enthalpy of thermal decomposition. DSC-IR is performed to detect decomposition products of CL-20/RDX. The results show that the decomposition products are mainly gases, such as CO_2_, N_2_O, and NO_2_. Finally, the impact sensitivity, friction sensitivity and thermal sensitivity of the samples were tested. The tests revealed that the mechanical sensitivity of CL-20/RDX is much lower than those of the raw materials and their blends and that the thermal sensitivity of CL-20/RDX does not decrease but increases.

## Conflicts of interest

There are no conflicts to declare.

## Supplementary Material
